# Performance analysis of English hospitals during the first and second waves of the coronavirus pandemic

**DOI:** 10.1007/s10729-023-09634-7

**Published:** 2023-05-09

**Authors:** Timo Kuosmanen, Yong Tan, Sheng Dai

**Affiliations:** 1 Department of Economics, Turku School of Economics, University of Turku, 20500 Turku, Finland; 2 School of Management, University of Bradford, Bradford, BD7 1DP West Yorkshire UK

**Keywords:** Convex regression, COVID-19, Healthcare management, Hospital performance

## Abstract

The coronavirus infection COVID-19 killed millions of people around the world in 2019-2022. Hospitals were in the forefront in the battle against the pandemic. This paper proposes a novel approach to assess the effectiveness of hospitals in saving lives. We empirically estimate the production function of COVID-19 deaths among hospital inpatients, applying Heckman’s two-stage approach to correct for the bias caused by a large number of zero-valued observations. We subsequently assess performance of hospitals based on regression residuals, incorporating contextual variables to convex quantile regression. Data of 187 hospitals in England over a 35-week period from April to December 2020 is divided in two sub-periods to compare the structural differences between the first and second waves of the pandemic. The results indicate significant performance improvement during the first wave, however, learning by doing was offset by the new mutated virus straits during the second wave. While the elderly patients were at significantly higher risk during the first wave, their expected mortality rate did not significantly differ from that of the general population during the second wave. Our most important empirical finding concerns large and systematic performance differences between individual hospitals: larger units proved more effective in saving lives, and hospitals in London had a lower mortality rate than the national average.

## Introduction

The coronavirus infection (henceforth COVID-19) started to spread from Wuhan, China, at the end of 2019 [1]. During the first half of 2020, it quickly spread all over the world and turned into a pandemic. By February 2022, almost six million people around the world had lost their lives to this disease.^1^ While there has been considerable research interest in COVID-19 in the literature of operational research and management science  [2–4], thus far there has been little attention on the performance of hospitals, which are in the forefront in the battle against the pandemic. Although human resources such as doctors and nurses and capital equipment such as motorized ventilation beds and protective masks are critically important, efficient organization and management of these resources is critical to save lives (cf., [5]). Identifying the best-performing hospitals and gaining better understanding of the factors that influence hospitals’ efficiency in dealing with the COVID-19 would be important for managerial and policy decisions to organize the hospital operations.

A large stream of operational research and management science literature considers efficiency, productivity and the returns to scale and scope in hospital operations [6–9]). While the present study is inspired by those previous studies on resource efficiency, this paper approaches hospital performance from a different perspective of *effectiveness of outcomes*. Instead of trying to model the production function of hospitals, which is a highly complex task with multiple parallel operations that interact creating spillovers [8], we consider a simpler task of modelling the COVID-19 mortality among hospital inpatients. We use the term “production function of death” to highlight the fact that we are not modelling the production function of hospitals, but indeed, that of the COVID-19 virus.^2^ We subsequently use the residuals of the empirical production function of death to assess performance of the COVID-19 care units.

Empirical implementation of the proposed conceptual idea involves at least two methodological challenges. The first challenge concerns the appropriate specification of the functional form of the production function and the modelling of contextual variables to capture the observed heterogeneity of hospitals and their operating environments. Building on the insights from the nonparametric frontier estimation literature [12, 13], the first methodological contribution is to extend the convex quantile regression [14–16] to incorporate contextual variables that characterize the operating environment of the COVID-19 care units. The quantiles provide a robust nonparametric approach to classify hospitals to performance groups (e.g., top 5%, 85-95%, $\cdots$, bottom 5%) without imposing any parametric distributional assumptions. In contrast to the context dependent data envelopment analysis [17] that iteratively peels off the best-performing units, the quantiles allow us to effectively utilize all data and control for the size of the performance groups. This paper combines the convex quantile regression with a partial linear model of contextual variables [12, 18] to allow the effects of operating environment differ across different levels of performances.

The second major challenge relates to the fact that approximately one half of the observations in the current study had zero COVID-19 related deaths. We would argue that the zero-valued outputs are a more significant problem than most empirical production studies recognize. In the estimation production functions, it is standard to apply logarithmic transformations $\textrm{ln}(y)$ to model heteroscedasticity with respect to size of the unit [18]. Since $\textrm{ln}(0)$ is undefined, the appropriate modelling of zero-valued observations forms a major methodological challenge in this context. A commonly used practical remedy is to add some small constant *c* to all observations (i.e., use $\textrm{ln}(y+c)$ [19]). However, we find that the regression results are highly sensitive to the specific choice of the constant *c*.^3^ To address this problem, we propose to exclude the zero-valued observations from the performance analysis, applying Heckman’s two-step estimator [20, 21] to correct for the resulting sample selection bias.

With more than 127 thousand confirmed COVID-19 deaths, more than 1,800 deaths per million inhabitants, the United Kingdom (UK) ranks among the most hard-hit nations in per capita terms. The empirical contribution of this study is to study the performance of English hospitals during the first and second waves of the COVID-19 pandemic using the unique data of the National Health Service (NHS) of England. More specifically, we focus on a 35-week period from April to December 2020 to investigate the performance of 187 hospitals in dealing with this virus, to shed light on the following empirical questions:

1. Were there systematic performance differences in the COVID-19 care at the regional or hospital levels?
2. Were there increasing or decreasing returns to scale in the COVID-19 care?
3. Did performance of COVID-19 care improve over time due to learning by doing?
4. Were there significant structural differences between the first and the second waves of the COVID-19 pandemic?

From the management point of view, the methodological and empirical results of this study could help the NHS and other national health service providers to develop systematic monitoring and benchmarking routines to identify and disseminate best practices more efficiently. In the event of possible future pandemics, more efficient identification and dissemination of best practices has potential to save thousands of lives.

The general approach developed in this paper could be readily applied in other countries and jurisdictions for which similar data are available at the hospital or regional level, not only in the context of the COVID-19 pandemic, but also for other hospital and health care operations such as emergency care or ambulance services. Indeed, the novel approach to performance analysis focusing on effectiveness of outcomes rather than resource efficiency forms the main contribution of this paper.

The structure of the paper will be organized as follows. Section 2 describes the empirical case and introduces the production function of death at the conceptual level. Section 3 introduces the theoretical model and the estimation approach, and proposes the methodological improvements. Section 4 presents the data sources and model variables. Section 5 and 6 present and discuss the results and managerial implications. Section 7 presents the concluding discussion. The GAMS code used in the computations is available on GitHub (https://github.com/ds2010/Covid-19).

## Empirical case

### COVID-19 epidemic in the UK

The first confirmed cases of COVID-19 in the UK were on the 29^th^ January 2020, when two Chinese nationals fell ill in York. The earliest known person to contract COVID-19 within the UK is believed to be a 75-year-old woman from Nottinghamshire, who tested positive on the 21^st^ February 2020 [22]. She is also understood to be the first victim of COVID-19 in the UK. In the end of February and during the first weeks of March 2020, both the daily number of positive cases as well as the daily death toll showed a dramatic increase, particularly in London.

After some initial hesitation, the UK government decided to issue a national lockdown, starting on the 23^rd^ March 2020. As a result, the epidemic started to slow down, and by the end of May there was a notable decline in the daily new positive cases and the death toll. The easing of the lockdown began on the 23^rd^ June. From the beginning of September, however, there was again a substantial increase in the number of daily positive cases. Fortunately, the mortality rate has been lower than the one of the first wave of the epidemic in the Spring 2020. During the second wave, the most affected areas include such cities as Liverpool, Manchester, Sheffield and Leeds in the Northern part of England. Since the number of COVID-19 cases was rising alarmingly, the government decided to issue another national lockdown on the 5^th^ November 2020. The NHS started its vaccination programme in December 2020, and more than 34 million had received the first dose by the end of April 2021. The first steps in easing the UK’s lockdown begun in March by allowing children to return to school. In England, all restrictions were lifted on the 19^th^ July 2021. However, travel restrictions and the mandatory use of face masks on public transport and at shops were reintroduced in November 2021 – January 2022 due to the wildly contagious Omicron variant.

The key objective of the lockdown and other restrictions introduced during the pandemic has been to slow down the spread of the COVID-19 virus in order to spare the hospital capacity to provide sufficient care to the COVID-19 infected patients and hence reduce the mortality of COVID-19 patients. Of course, the restrictions come at an enormous social cost, affecting not only the economic growth, but also human wellbeing in general. How effectively the hospitals can facilitate the recovery of the patients has a decisive impact on how harsh measures are required to slow down the spread of the Covid-19 virus.

### National Health Service

Established in 1948, the NHS is a governmental health care service that provides care to all the UK citizens: health care is tax financed and free at the point of use. The funding for health services in England comes from the department for Health and Social Care’s budget. The planned spending for 2021/22 was £190.3 billion, this included 33.8 billion extra funding in response to the COVID-19 pandemic.^4^ The NHS is one of the largest employers in the world, with 1.1 million full-time equivalent employees in England. In May 2021, there were 131,831 doctors, 346,582 nurses and health visitors (including midwives and health visitors), 35,256 managers out of total workforce of 1,193,666 (all figures are in full-time equivalent).

The NHS consists of a number of organizations that work both at the national and local levels. The Secretary of State for Health and Social Care is responsible for setting relevant policies for the NHS, including the waiting times, funding and staffing targets. Almost two thirds of the total NHS budget is controlled by the commissioning groups, which are run by general practitioners, nurses, and consultants. The commissioning groups have the responsibility of commissioning health care services for their local areas based on the assessed needs of the people, including primary care services, mental health, ambulance, social care, and hospital services. The budget of the commissioning groups is overseen by the NHS commission board, which has a number of regional offices around England. To promote competition, in 2006 the NHS mandated that all patients requiring treatment can choose between five different hospitals, and adopted a payment system in which hospitals are paid fixed, regulated prices for treating patients [23]. In 2012, the government introduced a series of further reforms to the NHS under the health and social care act, which gave greater freedom for the general practitioners to run the NHS budgets in their local area.

The care commissioned by the commissioning groups is provided by the NHS foundation trusts. As of April 2020, there are totally 219 foundation trusts. Each foundation trust is further divided into acute trust, mental health trust and ambulance trust. The responsibility of the acute trust is to make sure that they provide high-quality healthcare and resources are allocated in an efficient way. In comparison, the mental health trust is responsible for providing health and social care services to people with mental health problems. Finally, the ambulance trusts are mainly responsible for providing emergency access to healthcare.

### Modelling mortality of hospital inpatients

Since the early stages of the COVID-19 pandemic, there has been a lot of research on the mortality rate of this new virus  [24, 25]. It is important to draw a distinction between the mortality rate in the general population and among the hospital inpatients because many infected individuals get relatively mild symptoms and recover at home. Estimating the mortality rate among hospital inpatients is not as straightforward as it might appear. Ideally, one should systematically follow a cohort of patients admitted during a given time period until every patient of the cohort has either recovered or passed away, cf., [24]. Since the duration of hospital stay can vary from a few days to months, and since the criteria of admission vary across hospitals and between countries and jurisdictions, there is little comparable evidence about the mortality rate of COVID-19 among hospital inpatients.

To model the dynamics of the COVID-19 mortality among hospital inpatients, we adapt the notion of production function from economics as follows. Firstly, we consider the stock of inpatients as an input factor, analogous to the capital stock. We measure this input by the average number of beds occupied by the confirmed COVID-19 patients during a week, drawing a distinction between the regular hospital beds and the MV beds. The outflow of patients from the hospital can occur only through their discharge or death. Therefore, we consider the following two flow variables: i) the number of COVID-19 infected patients discharged from the hospital (weekly sum), and ii) the number of COVID-19 infected patients who died (weekly sum). The former can be regarded as a desirable output and the latter as an undesirable output. Note that in contrast to the input variables that measure the stock of inpatients during a given week, the outputs are flow variables that capture the outflow of inpatients from the stock. We therefore quantify the inputs by the weekly average, whereas the weekly sum is used as the flow variables.

The, NHS data also allows us to control for some contextual variables that represent the observed heterogeneity of hospitals and their operating environments. Most importantly, it is important to control for the share of senior patients in the stock of inpatients because the senior patients are known to be associated with a higher mortality rate when infected by COVID-19.^5^ To this end, we consider two contextual variables to capture the share of 65–84 year-old inpatients and the share of over 85 year-old inpatients. Since the NHS data does not report the bed occupancy statistics by age group, the shares of the two senior patient groups were approximated for each hospital in each week of the study period as the average share of senior inpatients among the new COVID-19 diagnoses and the admissions of COVID-19 diagnosed inpatients. In other words, we use the data of inflow of elderly patients to a hospital to approximate the stock of elderly inpatients in the hospital during a given week. Therefore, these two contextual variables capture the share of senior patients in the inflow to the inpatient stock.

Finally, we also want to control for the staff absence in the hospitals, which was a serious concern during the peak of the first wave of the pandemic (see, e.g.,  [26] for further discussion and evidence from Sweden). The NHS data reports the total number of staff members absent, but it is important to make this number proportionate to the total bed capacity of the hospital; absence of one nurse in a small hospital with ten nurses is a more serious problem than in a large hospital with hundreds of nurses. Assuming the hospitals were operating at or near their full capacity during the peak of the COVID-19 epidemic, we use the ratio of the total staff absence and the average weekly bed occupancy during the busiest week as our third contextual variable.

## Theoretical model and its estimation

### Production function of death

To operationalize conceptual model outlined in Section 2.3, consider the standard model of a single-output production function with contextual variables and stochastic noise [12, 13]

1 $$\begin{aligned} \textrm{ln} {y_{it}} = \textrm{ln} f(\textbf{x}_{it})+\mathbf {z'}_{it}\varvec{\updelta }+ \varepsilon _{it} \end{aligned}$$

where the subscripts refer to the production unit *i* in week *t*, output $y_{it}$ is the total number of deaths in the hospital *i* during week *t*. The vector of input variables $\textbf{x}_{it}$ includes the average occupancy of hospital beds by COVID-19 diagnosed patients ($x_{1it}$), and the average occupancy of motorized ventilation (MV) beds by the COVID-19 diagnosed patients ($x_{2it}$). The four contextual variables included are the share of 65–84 old patients ($z_{1it}$), the share of $+85$ old patients ($z_{2it}$), the ratio of staff absence to bed capacity ($z_{3it}$), and the time trend ($z_{4it} = t$). The production function *f* is assumed to be monotonic increasing and concave, but no specific functional form is assumed *a priori*. The random variable $\varepsilon _{it}$ is a composite error term that encompasses inefficiency and random noise, in other words, $\varepsilon _{it}$ does not necessarily have a zero mean or constant variance.

Note that the inputs of function *f* do not include the usual labour and capital inputs. To highlight the fact that *f* is not the hospital production function, we refer to function *f* as the *production function of death*. In contrast to the efficiency analysis, our main objective is not to isolate inefficiency from noise, but rather, examine properties of the production function *f* and the impacts of contextual variables $\varvec{\updelta }$ while recognizing that the empirical data are typically perturbed by inefficiency and noise. Note that the input variables $\textbf{x}$ must be scalable, ratio-scale measures, whereas the contextual variables $\textbf{z}$ are scale-invariant dummy variables, ratios or percentages.

### Quantiles and expectiles with contextual variables

To empirically estimate model (1), we propose to apply the shape-constrained semi-nonparametric regression subject to monotonicity and concavity constraints, also referred to as one-stage data envelopment analysis [18] or stochastic nonparametric envelopment of z-variables data [12]. To gain more insight on how the effects of the contextual variables interact with the level of performance, we also consider a more general approach referred to as convex quantile regression (CQR) [14, 16]. The first methodological contribution of this paper is to incorporate contextual variables to CQR.

More specifically, given the data generating process (1), the $\tau$^th^ conditional quantile of mortality, conditional on inputs $\textbf{x}$ and contextual variables $\textbf{z}$, is a function

$$\begin{aligned} Q(\tau \left| \textbf{x} ,\textbf{z} \right) = f^\tau (\textbf{x})\cdot \exp (\textbf{z}'\varvec{\updelta }^\tau + F_\varepsilon ^{-1}(\tau )) \end{aligned}$$

where $F_\varepsilon ^{-1}$ denotes the inverse cumulative distribution function of the error term $\varepsilon$ and $\tau \in (0, 1)$. The superscript $\tau$ is used to indicate that, in general, the $\tau$^th^ conditional quantiles of the production function *f* and the parameter vector $\varvec{\updelta }$ can differ across different levels of $\tau$. We have $f^\tau (\textbf{x})=f(\textbf{x})$ and $\varvec{\updelta }^\tau = \varvec{\updelta }$ for all $\tau \in (0, 1)$ if and only if the error term $\varepsilon$ is homoscedastic. Therefore, possible differences in the production function and the impacts of contextual variables across empirical quantiles can be attributed to heteroscedasticity of the error term $\varepsilon$. One potential source of heteroscedasticity is the presence of systematic performance differences across hospitals.

Several recent studies have developed methods to estimate the conditional quantile function $Q(\tau \, | \, \textbf{x})$ in the absence of contextual variables. Wang et al. [14] formulate a linear programming (LP) problem that makes use of monotonicity and concavity constraints developed for the convex regression [27]. However, solution to the LP problem is not necessarily unique, especially if the sample contains multiple observations with identical inputs. To guarantee a unique solution, Kuosmanen et al. [15] propose to resort to convex expectile regression (CER), which can be solved by quadratic programming. Recently, Kuosmanen and Zhou [16] propose an indirect approach to estimate quantiles by converting the estimated expectiles to the desired quantile, making use of the intimate connection between the quantiles and expectiles [28]. In this paper we resort to the indirect approach that employs CER to estimate the CQR model, and extend it to the log-transformed specification that allows us to introduce the contextual variables $\textbf{z}$ using a semi-nonparametric specification by Johnson and Kuosmanen [12].

For a given $\tau$, there exists a unique transfer function *h* such that $h(\tau ) = \tilde{\tau }$, where $\tilde{\tau }$ is the corresponding expectile [28]. For a given $\tilde{\tau }$, the CER estimator is defined as the optimal solution to the following asymmetric weighted least squares problem, which in the present case is a nonlinear programming (NLP) problem due to the logarithmic transformations:

2 $$\begin{aligned} \underset{\phi , \alpha , \varvec{\upbeta }, \varepsilon ^{+}, \varepsilon ^{-}}{\mathop {\min }}{} & {} \,\ (1-\tilde{\tau })\ \sum \limits _{i=1}^{n}{\sum \limits _{t=1}^{T}{{{(\varepsilon _{it}^{-})}^{2}}+\tilde{\tau }\sum \limits _{i=1}^{n}{\sum \limits _{t=1}^{T}{{{(\varepsilon _{it}^{+})}^{2}}}}}} \\ s.t. \quad{} & {} \ln y_{it} = \ln (\phi _{it}^{\tilde{\tau }}+1)+\textbf{z}'_{it}{{\varvec{\updelta }}^{{\tilde{\tau }}}}+\ \varepsilon _{it}^{+}-\varepsilon _{it}^{-} \qquad \forall i,\forall t \nonumber \\{} & {} \phi _{it}^{{\tilde{\tau }}}=\alpha _{it}^{{\tilde{\tau }}}+\textbf{x}'_{it}\varvec{\upbeta }_{it}^{{\tilde{\tau }}}-1 \quad \qquad \qquad \qquad \qquad \qquad \forall i,\forall t \nonumber \\{} & {} \alpha _{it}^{{\tilde{\tau }}}+\textbf{x}'_{it}\varvec{\upbeta }_{it}^{{\tilde{\tau }}} \le \alpha _{hs}^{{\tilde{\tau }}}+\textbf{x}'_{it}\varvec{\upbeta }_{hs}^{{\tilde{\tau }}} \qquad \qquad \qquad \qquad \forall i,h;\forall t,s \nonumber \\{} & {} \phi _{it}^{{\tilde{\tau }}}\ge 0\ \ \quad \quad \quad \quad \quad \quad \qquad \quad \quad \quad \quad \qquad \qquad \qquad \forall i,\forall t \nonumber \\{} & {} \textbf{0}\le \varvec{\upbeta }_{it}^{{\tilde{\tau }}}\le \textbf{1}\ \qquad \qquad \qquad \qquad \qquad \qquad \qquad \qquad \forall i,\forall t \nonumber \\{} & {} \varepsilon _{it}^{+}\ge 0,\ \varepsilon _{it}^{-}\ge 0\ \qquad \qquad \qquad \qquad \qquad \qquad \qquad \forall i,\forall t \nonumber \end{aligned}$$

where $\phi _{it}^{\tilde{\tau }}+1$ is the predicted value of the $f^{\tilde{\tau }}$,^6^ and $\varvec{\upbeta }_{it}^{\tilde{\tau }}$ are its gradient vectors in point $\textbf{x}_{it}$. The inequality constraints of problem (2) characterize $f^{\tilde{\tau }}$ as a piece-wise linear function that is monotonic increasing and concave [27]. Note that the standard convex nonparametric least squares (CNLS) estimator is the special case where $\tilde{\tau } = 0.5$, which is one appealing feature of the CER specification (2). In the present context, coefficients $\varvec{\upbeta }_{it}^{\tilde{\tau }}$ can be interpreted as the expected mortality rates of the COVID-19 patients in the regular beds and the MV beds, respectively. Since the mortality rate cannot be negative or greater than one, we restrict the coefficients $\varvec{\upbeta }_{it}^{\tilde{\tau }}$ to the closed interval [0, 1]. Similar constraints are widely used in the literature of data envelopment analysis, referred to as weight-restrictions or assurance regions.

We could adapt the direct quantile formulation by Wang et al. [14] to the present setting by replacing the objective function of (2) by

$$\begin{aligned} (1-\tau )\sum \limits _{i=1}^{n}{\sum \limits _{t=1}^{T}{\varepsilon _{it}^{-}+\tau \sum \limits _{i=1}^{n}{\sum \limits _{t=1}^{T}{\varepsilon _{it}^{+}}}}} \end{aligned}$$

Note that the resulting optimization problem must be solved using NLP due to the logarithm function in the first set of constraints. The main advantage of the indirect estimation of quantiles based on the CER regression of expectiles $\tilde{\tau }$ is to ensure uniqueness of the optimal solution. The estimated CER expectile $\tilde{\tau }$ can be subsequently converted to the corresponding quantile $\tau$ using the transfer function *h*; see Kuosmanen and Zhou [16] for a more detailed discussion. We will utilize the conversion of the estimated expectiles to the empirical quantiles in Section 5.3.

### Heckman correction of zero-valued observations

The NHS dataset (introduced in Section 4) includes a large number of observations where the output $y_{it}$ or the inputs $\mathbf {x_{it}}$ are equal to zero; approximately one half of the observations have $y_{it} = 0$. A commonly used practical remedy to avoid the logs of zeros is to add some constant *c* to all observations and use $\textrm{ln}(y+c)$ instead of $\textrm{ln}(y)$. Rocke and Durbin [19] refer to it as the “started logarithm”. Sometimes *c* is specified as a small number, say 0.0001, some studies set $c = 1$, but both choices are equally arbitrary. Unfortunately, the parameter estimates prove highly sensitive to the specific choice of the constant *c* used for the started logarithms to avoid the problem of zero values [10]. Recently, Ekwaru and Veugelers [29] propose to treat *c* in the $\textrm{ln}(y+c)$ as a model parameter, and jointly optimize it together with other model parameters. While this choice of constant *c* is not arbitrary, introducing an additional model parameter can contribute to overfitting.

Note that simply excluding the zero-valued observations would not only decrease the sample size, but also cause bias due to the truncation of the dependent variable. Suppose we simply exclude the zero-valued observations and estimate the model using the truncated data of $x > 0$. The logarithm function is now well-defined, but the truncation of the sample would cause bias. The situation is analogous to the sample selection bias examined by Heckman [20, 21]. Heckman’s key insight is to approach sample selection as a form of omitted-variables bias. Building on this insight, we propose to exclude the problematic zero-valued observations from the estimation, and correct for the resulting sample selection bias using the Heckman’s two-step estimator.

More specifically, the stepwise estimation procedure can be stated as follows:

- Step 1: Define the binary variable $Y_{it} = \{1$ if there occurs one or more COVID-19 related deaths in hospital *i* during week *t*, and 0 otherwise$\}$. Estimate the probit regression model
  $$\begin{aligned} Y_{it} = \Phi (\mathbf {x'}_{it}\varvec{\upgamma }+\textbf{z}'_{it}\varvec{\updelta })+\varepsilon _{it} \end{aligned}$$
  Given parameter estimates of $\varvec{\upgamma }$, $\varvec{\updelta }$, compute the inverse Mills ratios [30]
  $$\begin{aligned} IM_{it}=\phi (\textbf{x}'_{it}\varvec{\hat{\upgamma }}+\mathbf {z'}_{it}\varvec{\hat{\updelta }})/\Phi (\textbf{x}'_{it}\varvec{\hat{\upgamma }}+\mathbf {z'}_{it}\varvec{\hat{\updelta }}) \end{aligned}$$
  where $\phi$ and $\Phi$ denote the density function and the cumulative distribution function of the standard normal distribution *N*(0, 1).
- Step 2: Include $\text {IM}_{it}$ as one of the contextual variables $\textbf{z}$. For the sub-sample $D = \{i =1,\cdots , n; t=1, \cdots , T \, | \, Y_{it} = 1\}$ and expectile $\tilde{\tau }$, estimate the CER model (2). For the subsample *D*, predict the number of deaths in hospital *i* in week *t* by
  $$\begin{aligned} \hat{y}_{it}=(\hat{\alpha }_{it}^{{\tilde{\tau }}}+\textbf{x}'_{it}\varvec{\hat{\upbeta }}_{it}^{{\tilde{\tau }}})\cdot \exp (\textbf{z}'_{it}\varvec{\hat{\updelta }}_{{}}^{{\tilde{\tau }}}) \end{aligned}$$
  Relative performance of hospital *i* in week *t* can be measured in multiplicative form as
  $$\begin{aligned} Mperf_{it}=y_{it}/\hat{y}_{it} \end{aligned}$$
  or in the additive form as
  $$\begin{aligned} Aperf_{it}=y_{it}-\hat{y}_{it} \end{aligned}$$
- Step 3: For the subsample $O = \{i =1,\cdots ,n; t=1,\cdots ,T \, | \, Y_{it} = 0\}$, predict the number of deaths in hospital *i* in week *t* by using
  $$\begin{aligned} \hat{y}_{it}=\underset{h,s}{\mathop {\min }}\,(\hat{\alpha }_{hs}^{{\tilde{\tau }}}+\textbf{x}'_{it}\varvec{\hat{\upbeta }}_{hs}^{{\tilde{\tau }}})\exp (\textbf{z}'_{it}\varvec{\hat{\updelta }}_{{}}^{{\tilde{\tau }}}) \end{aligned}$$
  Subsequently, we can assess relative performance of hospital *i* in week *t* by
  $$\begin{aligned} Aperf_{it}=y_{it}-\hat{y}_{it} \end{aligned}$$
  Note that for the subsample *O* the multiplicative performance measure $Aperf_{it}$ is equal to zero by construction.

**Fig. 1:**
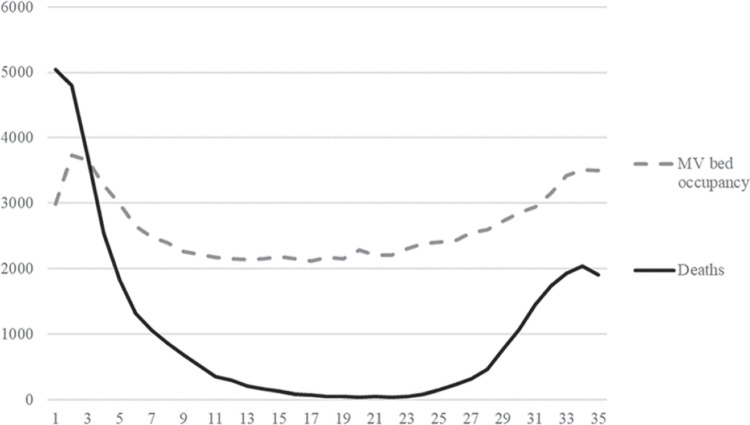
Development of the COVID-19 deaths and the motorized ventilation bed occupancy during the study period (weeks 1-35, from 2^nd^ April to 2^nd^ December 2020)

This procedure allows one to discard the problematic zero-valued observations in the estimation of production function without adding an arbitrary constant term. We correct for the resulting truncation bias by using the Heckman two-step procedure. Following Heckman, we use the probit regression in step 1, but one could alternatively apply the logistic regression (logit), or the panel data variants of probit or logit. Step 2 can be modified for estimating the convex regression, expectiles, or quantiles. One can also impose further properties such as constant returns to scale, or relax assumptions such as monotonicity or concavity [31]. Finally, the production function is estimated using the subset of data where $y_{it}$ is strictly positive, but for the purposes of performance assessment, the zero-valued observations should not be ignored: in the present setting, zero deaths is obviously the best possible outcome. Therefore, in Step 3, we compute the predicted number of deaths corresponding to those zero-valued observations. Note that the multiplicative performance indicator *MPerf* is equal to zero by construction, and hence, we propose to use the additive performance measure *APerf* for the zero-valued observations.

## Data

The data of the English hospitals over the period from the 2^nd^ of April 2020 to 2^nd^ December 2020 was obtained from the NHS website,^7^ covering the first and second waves of the pandemic. The NHS reports the hospital data at daily level, however, the daily variations are very large. To average out the arbitrary daily variations and weekday effects, we model the production function using weekly totals for the flow variables and weekly averages for the stock variables. Excluding hospitals with missing values, the final dataset includes 187 hospitals observed over a period of 35 weeks, which yields a balanced panel with 6545 observations. These hospitals are distributed across 7 different regions in England: East of England, London, Midlands, North East and Yorkshire, North West, South East and South West.

Table 1 presents the descriptive statistics of the inputs, outputs and contextual variables of the sample. We note that the average weekly number of regular beds occupied by the COVID-19 patients was almost ten times higher than the corresponding MV bed occupancy. The average number of deaths was 5.5 per week. Regarding to the contextual variables, we notice that the share of COVID-19 patients aged between 65 and 84 was on average 16% of the total COVID-19 patients, while the share of COVID-19 patients aged 85 or higher was on average 8%. Finally, we observe that the staff absence proportional to the bed capacity shows considerable differences across the hospitals in the sample.

To illustrate the development of the pandemic during the study period, Fig. 1 plots the weekly COVID-19 deaths and the MV bed occupancy during the study period. The sample period starts at the peak of the first wave of the pandemic in April when the weekly death toll was at its maximum level; unfortunately, hospital-level data prior to the 2^nd^ April are unavailable.

Figure 1 illustrates how the number of COVID-19 deaths started to rapidly decrease during the first ten weeks of the study period as the hospitals improved their operations and became more effective in saving lives of the COVID-19 infected patients. While throughout the summer of 2020, there were constantly more than 2,000 COVID-19 patients with severe symptoms treated in the MV beds, at best there were less than 50 deaths per week in July. Unfortunately, the death rate started to surge again since mid-September (week 25) as the virus had mutated to form new strains, which are considered to be more contagious.

To assess the possible structural changes between the first and the second waves of the pandemic, we partition the sample period in half at week 18 (30^th^ July- 5^th^ August), and refer to weeks 1-18 as the first wave, and weeks 18-35 as the second wave, respectively. This division is justified by two observations. First, the average MV bed occupancy reached its minimum level during the week 18, which is included in both sub-periods in our empirical analysis. Second, by comparing the two subperiods of equal length, we effectively avoid the possibility that differences in the sample sizes of the subperiods would add bias to our estimates.

**Table 1:** Summary statistics; sample size n = 6545

Variables	Mean	Std. Dev.	Min	Max
**Inputs (weekly averages)**				
Bed occupancy by COVID-19 patients	32.29	56.29	0	689
MV bed occupancy by COVID-19 patients	3.79	9.24	0	142
**Output (weekly sums)**				
Deaths of COVID-19 patients	5.51	12.58	0	218
**Contextual variables (ratios)**				
Share of 65–84 year-old COVID-19 patients	0.16	0.15	0	0.5
Share of over 85 year-old COVID-19 patients	0.08	0.11	0	0.5
Average staff absence per maximum bed occupancy	0.73	0.49	0	5.9

**Table 2:** Probit estimates; sample size n = 6545

	*Coefficient*	*Robust std. error*
Intercept	-2.007^***^	0.166
Bed occupancy	0.074^***^	0.005
MV bed occupancy	0.069^**^	0.030
Share of 65–84 year-olds	1.322^***^	0.143
Share of +85 year-olds	1.103^***^	0.196
Staff absence / max weekly bed occupancy	0.279^***^	0.096
Weekly time trend	-0.008^**^	0.003
London	reference category	
East	0.406^**^	0.178
Midlands	0.532^***^	0.158
North East	0.529^***^	0.175
North West	0.631^***^	0.191
South East	0.643^***^	0.179
South West	0.558^**^	0.166
Log pseudolikelihood	-2052.91	–

*** indicates statistical significant at 1% significance level, ** refers to 5% significance, * is 10% significance

## Results

### Probit regression

We first apply random-effects panel data probit regression to predict the probability of at least one death during the period of one week in all the 187 NHS hospitals in the sample. Table 2 reports the parameter estimates and their robust standard errors computed using the Stata 15 software package.

**Table 3:** CNLS estimates for the first wave, second wave, and the full study period

	*1*^*st*^ *wave*	*2*^*nd*^ *wave*	*Full period*
*Nonparametric part*	*Average estimates of* $\alpha _{it}, \varvec{\upbeta }_{it}$		
Intercept	1.132	1.015	0.968
Bed occupancy	0.093	0.079	0.081
MV bed occupancy	0.533	0.23	0.397
*Parametric part*	*Estimates of* $\varvec{\updelta }$		
Share of 65–84 year-olds	0.848^***^	0.057	0.274^***^
Share of +85 year-olds	0.849^***^	-0.399	0.068
Staff absence / max weekly bed occupancy	-0.048^*^	-0.059^*^	0.088^***^
Weekly time trend	-0.084^***^	0.002	-0.009^***^
London	reference category		
East	0.298^***^	0.247^***^	0.272^***^
Midlands	0.405^***^	0.235^***^	0.233^***^
North East	0.470^***^	0.317^***^	0.307^***^
North West	0.482^***^	0.338^***^	0.267^***^
South East	0.323^***^	0.215^***^	0.250^***^
South West	0.148^**^	0.141^**^	0.207^***^
Inverse Mills ratio	0.254^***^	-0.278^***^	-0.242^***^
$R^2$	0.786	0.782	0.759
Sample size	1938	1355	3260

*** indicates statistical significant at 1% significance level, ** refers to 5% significance, * is 10% significance

All the coefficients have the expected signs and are statistically significant. The probability of deaths occurring increases as the number of inpatients increases, if the share of elderly patients increases, or if there is staff absence. In contrast, the probability of death significantly decreases over time. We also include regional dummy variables choosing London as the reference category. Table 2 indicates that there are significant regional differences in the probability of deaths across regions.

The parameter estimates reported in Table 2 are used for computing the inverse Mills ratio for the subsample of 3260 observations in which the number of deaths was strictly positive. Including the inverse Mills ratio as an explanatory variable in the subsequent regression models corrects for the truncation bias caused by excluding the observations with zero deaths.

### Convex regression

Having excluded the zero-valued observations and included the inverse Mills ratio as a contextual variable, we next apply the CNLS estimator, which is the special case of the CER formulation (2) obtained by setting $\tilde{\tau } = 0.5$. We divide the sample period into two parts, representing the first and the second waves of the pandemic (weeks 1–18 and 18–35, respectively), but we also consider the entire study period (weeks 1–35). The resulting NLP problems were solved using GAMS/KNITRO (12.2.2) on Aalto University’s high-performance computing cluster Triton with Xeon @2.8 GHz processors, 13 CPUs, 80GB RAM per CPU; the GAMS code used in the computations is available on GitHub (https://github.com/ds2010/Covid-19).

Table 3 summarizes the CNLS results. The top part of the table reports the average values of the observation-specific coefficients that characterize the nonparametric production function *f*. The bottom part reports the regression coefficients of the contextual variables $\textbf{z}$ modelled in a parametric fashion. The bottom row of the table reports the coefficient of determination $R^2$.

Recall that coefficients $\varvec{\upbeta }_{it}$ differ across the observations (Table 3 reports average estimates), and represent the expected mortality rates of inpatients. We find a notable decrease in the expected mortality from the first wave to the second one, especially for the patients treated in the MV beds. Since the MV bed occupants are typically patients with the most severe COVID-19 symptoms, the decrease in the expected mortality rate from 0.53 to 0.23 is quite remarkable, increasing the probability of survival of average patient in the MV bed from less than 50% to more than 75%.

The estimated intercepts $\alpha _{it}$ are systematically greater than zero for all the observations, which indicates decreasing returns to scale. In the present context, the decreasing returns imply that the larger units have been more effective in saving lives of the COVID-19 patients, consistent with the previous empirical literature on hospital efficiency [8]. This would suggest that the hospitals that care for a larger number of COVID-19 patients appear to be more effective in avoiding death. However, we cannot tell if the number of COVID-19 patients correlates with the size of the hospital, or whether the size of the hospital has a direct effect on the mortality rate (e.g., larger hospitals may have better equipment, personnel, procedures or management). There may be several kinds of selection effects in play, for example, the most efficient hospitals might be assigned larger numbers of patients, or the patients with the most severe symptoms could be isolated to the smaller units. The underlying sources of the significant economies of scale found in this study would clearly warrant further investigation.

The estimated coefficients of the contextual variables also reveal interesting patterns. During the first wave, the elderly inpatients of age 65–84 years or more than 85 years were at significantly higher risk of dying than the younger patients. During the second wave, the shares of elderly inpatients are no longer significant predictors of death. Possible explanations include the improved hospital practices, but also the mutated straits of COVID-19 that are considered to be more contagious among younger people.

We find a significant negative time trend during the first wave. The absolute value of the time trend coefficient is relatively large, which points to rapid learning by doing during this period (see, e.g., [32] for an insightful discussion of the role of learning by doing in productivity growth). Interestingly, the time trend is no longer significant during the second wave.

Staff absence has a significant positive effect on COVID-19 deaths according to the pooled model that includes the entire study period. Somewhat surprisingly, the signs of the staff absence coefficient turn to negative when we only consider the two sub-periods representing the first and the second wave of the pandemic. However, these negative coefficients are statistically insignificant and small in magnitude.

Finally, we apply regional dummy variables to test if there were significant performance differences across regions. The reference category is London, which is the best-performing region. All the other NHS regions had significantly higher expected mortality rates. Applying the exponential function to the coefficients of the regional dummy variables (e.g., exp(0.482) = 1.62), we find that during the first wave in the North East and North West, the expected mortality rate was more than 60% higher than in London, keeping all the other factors constant. The comparison of the two subsamples representing the first and the second waves indicates a positive result that the performance gaps to London decreased over time, but the North East and North West had still the highest expected mortality rates.

The best performance of the London hospitals is not surprising as such, given the large number of hospitals in close proximity to one another, and the high concentration of top hospitals and physicians, cf., [23]. However, the large performance gaps to other regions are alarmingly large, and call for an explanation. Although our analysis is based on hospital-level data, we must stress that the regional organization of the health care system can also influence the performance gaps. For example, inadequate ambulance services at regional level can cause delay in the admission to hospital, which may result as higher mortality rate of COVID-19 inpatients, and show as poor hospital performance in our analysis. Thus, the observed performance differences cannot be solely attributed to the hospital management, the organization and management of the health care system at the regional level can also affect hospital performance. Moreover, differences in the population, including the age distribution and comorbidities such as diabetes, obesity, or kidney disease, may also contribute to performance differences across hospitals. While the role of comorbidities could be significant at the hospital level, at the aggregate level of regions, individual and local differences in comorbidities tend to cancel out. The observed performance gaps at the regional level are so large that regional differences in comorbidities seems a highly unlikely explanation. We stress that the main contribution of the present paper is to provide evidence that such performance gaps existed. To avoid excess mortality in possible future pandemics, it would be important to investigate the underlying reasons behind such large regional differences in the expected mortality of COVID-19 inpatients more thoroughly, but such an investigation falls beyond the scope of the present study.

### Expectiles and quantiles

To gain deeper understanding of the possible structural differences between the best performing hospitals and the weakest performers, we next apply the convex expectile regression formulation (2) with six alternative parameter values $\tilde{\tau }=$ 0.05, 0.15, 0.25, 0.75, 0.85, and 0.95. We are particularly interested in whether the estimated coefficients of the contextual variables $\textbf{z}$ differ across different expectiles. Recall that the differences in these parameter estimates across different expectiles reflect heteroscedasticity of the composite error term $\varepsilon$ with respect to the contextual variables $\textbf{z}$: if $\varepsilon$ is homoscedastic with respect to a given contextual variable, then the CER estimates of the corresponding $\varvec{\updelta }$ should be relatively constant across expectiles. Further, since heteroscedasticity of $\varepsilon$ is associated with asymmetric performance differences, possible differences in the effects of the contextual variables across expectiles can shed light on the underlying drivers of performance differences.

Table 4 reports the parameter estimates for the six quantiles noted above, which in the present context represent the best performance and the worst performance, respectively.^8^ Recall that the case of the average performance $\tilde{\tau }= 0.5$ was already considered in the previous sub-section. To gain further intuition, we have also converted the expectiles to the corresponding empirical quantiles $\tau$ using the empirical strategy suggested by Efron [33]. For example, Table 4 indicates that the quantile $\tilde{\tau }= 0.05$ corresponds approximately to the quantile $\tau = 0.12$, which means that the left-most column of Table 4 refers to the coefficients of the best-performing decile of observations. Further, the weakest performance in this comparison, $\tilde{\tau }= 0.95$, corresponds to the quantile $\tau = 0.76$; in other words, the right-most column of Table 4 refers to the bottom quartile of observations in terms of performance. The main purpose of Table 4 is to illustrate how the effects of the contextual variables depend on the level of performance.

**Table 4:** Estimates of the contextual variables for the selected expectiles/quantiles, the full study period (weeks 1-35, 3260 observations)

	*Best performance*				*Worst performance*		
*Expectile*	$\tilde{\tau }=0.05$	$\tilde{\tau }=0.15$	$\tilde{\tau }=0.25$	$\cdots$	$\tilde{\tau }=0.75$	$\tilde{\tau }=0.85$	$\tilde{\tau }=0.95$
*=Quantile*	$\tau =0.12$	$\tau =0.21$	$\tau =0.27$	$\cdots$	$\tau =0.56$	$\tau =0.64$	$\tau =0.76$
Share of 65–84 year-olds	-0.063	0.106	0.17		0.326	0.326	0.344
Share of +85 year-olds	-0.156	-0.032	0.004		0.09	0.073	0.015
Staff absence /	0.016	0.043	0.063		0.1	0.098	0.091
max weekly bed occupancy							
Weekly time trend	-0.009	-0.009	-0.009		-0.01	-0.011	-0.013
London	reference category						
East	0.178	0.235	0.251		0.269	0.267	0.271
Midlands	0.164	0.226	0.234		0.224	0.217	0.203
North East	0.202	0.275	0.293		0.308	0.307	0.31
North West	0.187	0.238	0.252		0.27	0.274	0.286
South East	0.153	0.219	0.238		0.241	0.235	0.225
South West	0.16	0.196	0.207		0.196	0.185	0.16
Inverse Mills ratio	-0.282	-0.306	-0.297		-0.158	-0.102	0.013

Consider first the shares of the elderly inpatients, the coefficients of which are reported on the first two rows of Table 4. Interestingly, the elderly inpatients had a slightly lower expected mortality rate in the top decile of observations (the left-most columns of Table 4), but unfortunately, the expected mortality rates of the elderly increase as we move to the bottom quartile of observations, especially for the group of 65–84 year-old patients. Note that a hospital may rank in the top decile during some weeks, but fall to the quantiles during other weeks. This is why we refer to the top decile of observations rather than specific hospitals.

Staff absence has a positive effect on expected mortality, but its impact is considerably lower among the best performing observations than in the worst ones. The weekly time trend has a relatively stable negative effect across all expectiles, which suggests relatively homogenous rate of learning by doing. However, the worst performers have a slightly smaller coefficient, which may be associated with catching up the best performing units.

Considering the regional performance differences, Table 4 highlights the fact that the regional differences are much more pronounced among the worst performers than among the best performing hospitals. This would support the hypothesis that performance differences likely occur at the hospital level: the best performing hospitals just happen to be located in London and the worst performers are in the North East and North West. The observed regional differences just reflect differences in the average performance of the hospitals located in those specific regions, but there are not necessarily any inherent regional differences in the population, the hospital management, or the COVID-19 virus itself.

## Managerial implications

When the first cohorts of COVID-19 infected patients were admitted to hospitals, all medical teams were inexperienced in the care of this unprecedented disease. During the first wave of the pandemic, our results indicate a sharp decrease in the expected mortality of COVID-19 inpatients: mortality decreased 8% per week on average based on the estimated time trend. This impressive performance improvement can be attributed to learning by doing. Unfortunately, in the absence of systematic performance management and benchmarking, there was a large gap between the best performing and the weakest performing hospitals. Presence of such large performance gaps, both at the regional and hospital levels, would suggest that inefficient practices and slow diffusion of information caused loss of life that could had been avoidable by better management. The empirical results of our study have three key managerial implications with a view towards more effective management of possible future pandemics, but also to better management of other hospital operations such as emergency care or ambulance services.

First, the performance assessment approach proposed in this study would enable NHS and similar health care organizations around the world to monitor hospital performance virtually in real time, utilizing the data that is already collected and published by NHS. Identifying the best performing hospitals forms the first step of best practice benchmarking, which could help medical teams to identify specific practices that help to save lives of COVID-19 inpatients, and disseminate information about these practices across all hospitals. During the first weeks of the COVID-19 pandemic, medical teams gained valuable experience on how to best utilize the MV bed capacity and in which position to lay the COVID-19 inpatients in hospital beds.^9^ Systematic performance monitoring and benchmarking could help to identify such valuable information on best practices quicker, and disseminate it more systematically across hospitals. Systematic performance monitoring and benchmarking would also create strong incentive to the hospital managers, in particular to the ones that were not performed very well in this pandemic, to further improve and develop their management practices. While public reporting and transparency of hospital performance in COVID-19 care might help to incentivize the hospitals for better performance, cf., [34], we would attribute the performance gaps identified to inefficient policy and management at the higher level of the health care system (cf., [35]). Establishing performance monitoring and benchmarking systems is our first managerial lesson with a view towards the future pandemics.

Second, our results show that larger COVID-19 care units were more effective in saving lives of COVID-19 patients than smaller units. While the volume-outcome effect on mortality is well-established in the literature [36], to our knowledge, the economies of scale in terms of the reduced COVID-19 mortality is a new finding. While the underlying factors behind the economies of scale in the COVID-19 care would warrant further research, we suspect that superior performance of larger units may relate to more efficient capacity utilization, better team work by the medical professionals, as well as quicker dissemination of best practices. Economies of scale could also relate to spillover effects between COVID-19 care and other hospital operations, cf., [8], however, it is worth to emphasize that the size of the COVID-19 unit is not necessarily perfectly correlated with size of the hospital. The key managerial lesson is to try to allocate COVID-19 patients to larger units where feasible.

Third, we find large systematic regional differences in hospital performance, both during the first and the second waves of the pandemic. In particular, expected mortality of COVID-19 patients was significantly lower in London hospitals than in all other regions. Even after controlling for the size of the COVID-19 unit, the share of senior patients, and staff absence, hospitals in the North East and North West had 60% higher expected mortality rate than London hospitals during the first wave of the pandemic, decreasing to 40% during the second wave. Such large regional performance differences indicate significant inequalities between the patients hospitalized in different NHS regions. These results also suggest that more efficient identification and dissemination of information on best practices from the best performing London hospitals to the other NHS regions might had saved thousands of lives. While the significant performance differences during the first wave of the pandemic were to some extent unavoidable, there was sufficient time to identify best practices and disseminate information prior to the second wave of the pandemic. While the reforms of NHS over the past decade have given greater freedom and independence for the local authorities, the policy reform may have reduced coordination and oversight at the national level.

In conclusion, based on our empirical results we would encourage NHS and other national health service providers to 1) establish systematic performance monitoring and benchmarking procedures to identify and disseminate best practices; 2) utilize economies of scale by allocating patients to larger units when feasible; and 3) ensure sufficient coordination between the regional care providers to facilitate more efficient dissemination of best practices not only locally but nationwide.

## Conclusions

Hospitals around the world were in the forefront in the battle against the COVID-19 pandemic, however, thus far there has been little attention on the performance of hospitals in saving lives. This study proposed to assess performance from a novel perspective, introducing the conceptual notion of the production function of death that approaches hospital performance from the perspective of the effectiveness of outcomes instead of the conventional notions of efficiency and productivity.

To apply the proposed approach to the empirical data, this study addressed two methodological challenges. First, we incorporated contextual variables to the convex quantile regression to gain further insights on the impacts of the contextual variables at different levels of performance. Second, we developed a theoretically new approach to model the zero-valued observations, making use of Heckman’s two-step approach to correct for the sample selection bias. While the zero-valued outputs present a major challenge in this specific application, we would argue that the zero-valued outputs are rather common in the empirical studies and that inappropriate modelling of zero outputs can cause serious bias in the empirical results. The two-stage bias correction proposed in this study could be readily applied in other production studies to alleviate such bias.

Our empirical findings reveal a significant negative trend in the production function of death during the first wave of the pandemic. We also find significant decreasing returns to scale, which implies that the larger COVID-19 units are more effective in saving lives. The mortality rate of inpatients is also significantly and positively associated with the share of senior patients aged 65 and above. Comparing the hospital performance among different areas of England, we find that the hospitals in London had lower mortality than the national average, while the ones in the North East and North West showed weakest performance. Finally, there are large and systematic performance differences between individual hospitals, which would warrant further investigation. We found the quantile approach a useful complementary tool to gain deeper insight on the structural differences between the best performing and the worst performing hospitals. In particular, the best performing hospitals excelled in saving lives of elderly patients, but also managed to cope with staff absence better than the worst performing hospitals. Interestingly, the regional performance differences were also more pronounced for the worst performing hospitals. This seems to suggest that the performance differences may actually occur at the hospital level: there are not necessarily any inherent regional differences, however, the best performing hospitals are located in London. Our empirical analysis revealed several structural differences between the first and the second waves of the pandemic in England. It is fortunate that the expected mortality rates have decreased notably over time, especially for the elderly inpatients and those with the most severe symptoms. The doctors, nurses and other hospital staff have demonstrated impressive ability to improve performance through learning by doing. Unfortunately, there are significant performance differences both across the NHS regions and individual hospitals, which cannot be explained by the staff absence or the share of senior patients.

We hope that the findings of this line of research could help the NHS to identify and disseminate more broadly the best hospital practices in saving lives. After the study period considered in this paper, the daily number of the positive COVID-19 cases kept increasing, peaking at more than 68 thousand on the 8^th^ January 2021. As a result, the UK issued another national lockdown from the 6^th^ January 2021 to March 2021. The NHS started its COVID-19 vaccination programme on the 8^th^ December 2020. The approved vaccines are expected to be effective against the UK strain of COVID-19, but while writing this, it is too early to declare the battle against COVID-19 to be over at the global level.

Considering possible future pandemics, it would be critically important to gain better understanding of the factors influencing hospital performance during the COVID-19 pandemic to be better prepared when another unprecedented virus appears. While our empirical analysis focused on the NHS hospitals in England, the general approach developed in this paper could be readily applied to other countries and jurisdictions for which similar data are available at the hospital or regional level. While the approach has been developed in the context of the COVID-19 pandemic, it is more broadly applicable to analysing performance of hospitals or other healthcare providers in saving lives, for example, in emergency care or ambulance services.

## Footnotes

1 Source: https://covid19.who.int/

2 This article synthesizes the conceptual idea, methodological developments, and the empirical insights of the two working papers [10, 11] written during height of the COVID-19 pandemic in 2020.

3 See the working paper Kuosmanen et al. [10] for more detailed discussion and evidence.

4 Source: https://www.kingsfund.org.uk/projects/nhs-in-a-nutshell/nhs-budget

5 The question of causality is immaterial for performance comparison of hospitals, which is the main objective of the present study. For example, the age and mortality are positively correlated, but we do not make any claims about their causal relationship.

6 We add the value of one to ensure that the logarithm function is well-defined, but subtract it in the next constraint.

7 https://www.england.nhs.uk/statistics/statistical-work-areas/covid-19-hospital-activity/ and https://www.england.nhs.uk/statistics/statistical-work-areas/covid-19-daily-deaths/ (downloaded on 16^th^ December 2020).

8 Normally the best performance would be associated with the highest values of $\tau$, but since we here estimate the production function of death, saving lives of inpatients requires making COVID-19 as inefficient as possible.

9 NHS: Coronavirus - how lying on your front can help get more oxygen into your body. Retrieved from: https://www.wsh.nhs.uk/CMS-Documents/Patient-leaflets/Coronavirus/6628-1-Coronavirus-how-lying-on-your-front-to-can-help-get-more-oxygen-into-your-body.pdf.
